# Beyond the Total Score: Revealing Cognitive Heterogeneity in High Ability Through Latent Profile Analysis

**DOI:** 10.3390/jintelligence14080166

**Published:** 2026-08-01

**Authors:** Norah Almulhim, Abdullah M. Aljughaiman, Fahad S. Alfaiz

**Affiliations:** 1 Special Education Department, King Faisal University, Al-Ahsa 36362, Saudi Arabia; alju9390@gmail.com; 2 Self-Development Skills Department, Common First Year Deanship, King Saud University, Riyadh 11451, Saudi Arabia; falfaiz@ksu.edu.sa

**Keywords:** latent profile analysis, cognitive heterogeneity, gifted identification, cutoff scores, multidimensional assessment

## Abstract

This study examined whether a single total-score cutoff in the Mawhiba Multiple Cognitive Aptitude Test (MMCAT) may conceal meaningful cognitive profile differences between students across four domains: mental flexibility, verbal reasoning and reading comprehension, mathematical–spatial reasoning, and scientific–mechanical reasoning. Archival score data from 5146 school-nominated students across three assessment levels in Al-Ahsa, Saudi Arabia, were analyzed. Latent profile models were estimated separately for each assessment level. A three-profile solution was retained for Levels 1 and 2, and a four-profile solution for Level 3. The retained profiles differed mainly in the overall performance level rather than the profile shape. The operational cutoff of 1540 captured globally high-performing profiles at considerably higher rates than adjacent middle-range profiles, and the identified groups remained internally heterogeneous across all levels. The findings indicate that the current identification rule effectively captures broad, high-level performance, yet it does not represent all adjacent cognitive profiles equally. This has implications for identification policy and educational programming for gifted students.

## 1. Introduction

Giftedness is now widely understood as a multidimensional construct comprising diverse cognitive abilities rather than a single measure of general intelligence (Samsen-Bronsveld et al., 2024; Sternberg & Kaufman, 2018). However, despite the recognition of the multidimensional nature of giftedness, overall cognitive scores continue to play a prominent role in gifted identification and placement decisions (Silverman & Gilman, 2020). Nevertheless, research suggests that while overall scores demonstrate strong statistical efficiency, they may obscure meaningful variation between an individual’s underlying cognitive abilities (Pezzuti et al., 2022).

Research suggests that gifted individuals do not form a homogeneous group but rather differ substantially in their cognitive profiles and patterns of abilities (Silverman, 2009). Consequently, there is a growing need to move beyond traditional variable-centered approaches and adopt person-centered methods, such as latent profile analysis. These methods allow researchers to identify distinct cognitive patterns within groups that may appear homogeneous at first glance and reveal how abilities are distributed within students rather than relying solely on overall performance (Öpengin & Bal Sezerel, 2023).

Despite these theoretical and empirical arguments, significant gaps remain in the literature. It is unclear whether the cutoff scores used in gifted identification systems are equally sensitive to different cognitive profiles or whether they favor the identification of certain patterns over others. Little is known about how many cognitive profiles exist within identified gifted populations or how these profiles differ from one another. It also remains unknown whether the heterogeneity observed within identified groups reflects meaningful differences in the cognitive profile shape or whether it primarily reflects differences in the overall performance level. To address these gaps, the present study applied latent profile analysis to subscale scores from the Mawhiba Multiple Cognitive Aptitude Test (MMCAT) to identify latent cognitive profiles among gifted students and examine whether a single total-score cutoff conceals meaningful variation in cognitive performance.

## 2. Literature Review

### 2.1. The Cognitive Profile of Intellectual Giftedness

Traditionally, giftedness has been defined in terms of Intelligence Quotient (IQ) scores obtained from standardized tests, often reducing giftedness to a single numerical indicator of general cognitive ability (Stoeger, 2009). However, theories of intelligence increasingly emphasize its multidimensional nature. Carroll’s (1993) three-stratum theory, Gardner’s (1983) theory of multiple intelligences, and Sternberg’s (1985) triarchic theory differ in their assumptions but broaden the understanding of giftedness beyond general intelligence alone by emphasizing multiple cognitive abilities. This shift reflects increasing evidence that giftedness cannot be fully explained by a single dimension (Davidson, 2009).

Emerging evidence further supports this view, suggesting that psychometrically defined intelligence becomes increasingly differentiated at the upper end of the ability distribution (Blum & Holling, 2017). As a result, two individuals with the same overall score may exhibit markedly different cognitive profiles (Dai, 2009). For example, two students may achieve the same overall score while showing very different patterns of strengths and weaknesses across specific cognitive abilities. One student may excel in analytical abilities, while the other may demonstrate stronger creative or linguistic abilities.

Despite growing recognition that cognitive ability is multidimensional, overall test scores and cutoff criteria remain central components of many gifted identification systems. These cutoffs do not represent natural boundaries in the structure of cognitive ability, but rather reflect policy decisions shaped by technical, empirical, and value-based factors (AERA et al., 2014). Research suggests that gifted students tend to show markedly uneven cognitive profiles, with strong verbal comprehension and perceptual reasoning skills alongside lower performance in working memory and processing speed, a pattern that the overall score obscures rather than reflects (Pezzuti et al., 2022). Furthermore, Peters et al. (2023) argue that the quality of identification systems should be evaluated by their ability to identify all students who genuinely qualify for services, as well as by their efficiency. Taken together, these findings raise questions about whether a single overall score can adequately capture the variation observed between gifted students.

### 2.2. The Person-Centered Approach in Gifted Research

One of the most prominent statistical methods for identifying such patterns is latent profile analysis (LPA), which aims to identify internally homogeneous and mutually distinct subgroups based on patterns of responses across multiple variables (Mammadov et al., 2016; Spurk et al., 2020). Unlike traditional variable-centered methods, such as factor analysis and regression, which focus on relationships between variables, LPA adopts a person-centered approach that focuses on patterns of characteristics within individuals.

Latent profile analysis has been used in giftedness research to identify heterogeneity within gifted populations. In the field of cognitive classification specifically, Öpengin and Bal Sezerel (2023) used this method on data from 360 gifted students who had achieved a score of 130 or higher on the Anadolu–Sak Intelligence Scale. The analysis yielded two distinct cognitive profiles: verbally gifted (38%) and non-verbally gifted (62%). These findings challenge the assumption that gifted populations are cognitively homogeneous and suggest that multiple ability patterns should be considered in identification and programming decisions. This approach has also been extended to studying personality profiles (Mammadov, 2023) and motivational orientation (Kang, 2021) among gifted adolescents, as well as self-regulated learning skills (Gómez et al., 2026) and ability and psychological competence in talent development programs (Mahatmya et al., 2023). However, the application of LPA to the subscale scores of multidimensional gifted identification instruments and its use in evaluating the sensitivity of operational cutoff scores remain limited.

### 2.3. Purpose of This Study

This study examined whether the use of a single total-score cutoff in the MMCAT may hide meaningful differences in students’ cognitive profiles. The analysis focused on four domains in the scale: mental flexibility, verbal reasoning and reading comprehension, mathematical–spatial reasoning, and scientific–mechanical reasoning. Instead of assuming that students with similar total scores also have similar patterns of strengths and weaknesses, the study asked whether distinct latent profiles could be identified in the screened sample, whether those profiles mainly reflected differences in the overall level of performance or some difference in profile shape, and how well the operational cutoff of 1540 captured the retained profiles.

### 2.4. Research Questions

1. Does the data show hidden cognitive heterogeneity?
2. How evenly does the 1540 cutoff capture the retained profiles?
3. Do profile distributions vary across background variables?

## 3. Materials and Methods

### 3.1. Research Design

This study used a cross-sectional secondary-data design based on archival records from the 2026 administration of the MMCAT in Al-Ahsa, Saudi Arabia. The main question was whether one composite cutoff score may hide meaningful variation in students’ multidimensional performance. To answer this question, profile discovery came before cutoff evaluation. Latent profiles were first identified in the full level-specific screened sample within each assessment level, and the operational cutoff of 1540 was then used afterward to examine how often the current identification rule captured each retained profile.

### 3.2. Participants

The broader student population in Al-Ahsa is estimated at about 260,000 students. The analytic dataset included 5146 student records from the 2026 screening cycle. Therefore, this dataset should be understood as a screened administrative sample, not a random sample from the full student population.

These data came from the official process used to identify students for gifted programs in Saudi Arabia. Each year, schools are invited to nominate students for gifted programs by using standardized nomination forms. Only students who are nominated by their schools are allowed to take the MMCAT. The test is administered centrally in major cities across the Kingdom. For this reason, the present dataset represents students who passed through the official school nomination and screening process, not all students in Al-Ahsa.

The dataset included 3111 females (60.5%) and 2035 males (39.5%). Students came from eight grade levels: Grade 3 (*n* = 789, 15.3%), Grade 4 (*n* = 392, 7.6%), Grade 5 (*n* = 358, 7.0%), Grade 6 (*n* = 1264, 24.6%), Grade 7 (*n* = 301, 5.8%), Grade 8 (*n* = 310, 6.0%), Grade 9 (*n* = 1298, 25.2%), and Grade 10 (*n* = 434, 8.4%). Students attended public schools (*n* = 4114, 79.9%), private schools (*n* = 543, 10.6%), and international or foreign schools (*n* = 474, 9.2%). The school type was missing for 15 cases (0.3%). Most students took the Arabic version of the assessment (*n* = 4882, 94.9%), while 264 students (5.1%) took the English version.

The dataset also included three official assessment levels. These levels matched different grade bands. Level 1 included Grades 3–5 (*n* = 1539, 29.9%), Level 2 included Grades 6–8 (*n* = 1875, 36.4%), and Level 3 included Grades 9–10 (*n* = 1732, 33.7%). Using the official total-score cutoff of 1540, a total of 1523 students (29.6%) met or exceeded the identification threshold. This cutoff-defined subgroup was not used as a separate estimation sample for the latent profiles; rather, the latent profiles were estimated in the full level-specific screened sample, and the cutoff-defined subgroup was used afterward only to describe the proportions of students above versus below the cutoff within each retained profile.

### 3.3. Measurements

The data included one total score for the MMCAT, along with its four standardized domain scores: mental flexibility, verbal reasoning and reading comprehension, mathematical–spatial reasoning, and scientific–mechanical reasoning. The MMCAT has shown strong psychometric properties. Evidence from its development studies supported its reliability and validity, including exploratory and confirmatory factor analyses that confirmed its four-domain structure. The test was also standardized on a nationally representative Saudi sample, and technical studies demonstrated its ability to distinguish between different levels of ability across the four domains (National Center for Assessment, 2018). Subsequent studies further examined its psychometric properties at scale, providing additional construct validity evidence through latent class and factor-analytic approaches (Dimitrov & Alharbi, 2014; Alharbi & Dimitrov, 2015).

Higher scores reflected stronger performance. The total MMCAT score was the administrative score used for identification, and 1540 was the official cutoff in the 2026 cycle. The data also included gender, grade, school type, assessment level, test language, and the operational giftedness classification assigned by the system. Item-level responses and the technical manual were not available for the present study. Therefore, the study did not re-estimate the reliability or test item-level measurement models. Instead, the domain scores were used as official standardized outputs of the assessment system and were interpreted as indicators of students’ relative standing rather than raw scores. The available documentation did not provide enough information to determine how the standardized domain scores were constructed or whether they were directly comparable across assessment levels, gender, school type, or test language (Arabic versus English). For this reason, all profile analyses were conducted separately within each assessment level.

### 3.4. Data Preparation

The original data included direct student identifiers. These identifiers were removed before the analysis. The final dataset kept only the variables needed for the study. The total score and the four domain scores were complete for all cases used in the main analyses. Missing data were very limited and were found mainly regarding school type. Because the variables used in the latent profile analysis were complete, no data imputation was needed.

### 3.5. Data Analysis

All profile analyses were conducted separately by assessment level because level and grade band were structurally linked. As a preliminary step, descriptive statistics and correlations were computed for the total score and four domain scores within each level.

To answer the first research question on whether the data showed hidden cognitive heterogeneity, latent profile models were estimated within each level using Gaussian finite-mixture models under a local-independence specification (diagonal within-profile covariance matrices) and a maximum-likelihood expectation-maximization routine. All mixture models were estimated in Python 3.13 using scikit-learn 1.8.0. Models with one to six profiles were estimated for each level. To improve the solution stability, each model was estimated from 100 k-means-based starting values, and the highest log-likelihood solution was retained. Across repeated random-start estimations, the retained models returned the same substantive profile pattern, and the final interpretation was also checked against alternative covariance structures.

Because the implementation used for this study did not allow for a Lo–Mendell–Rubin or bootstrap likelihood-ratio test, profile retention relied on the AIC, BIC, entropy, average posterior assignment probability (APPA), smallest class size, substantive interpretability, and parsimony. Although some later solutions yielded modestly lower information criteria, inspection of the profile means and cross-structure re-estimation showed that those additional classes mainly subdivided adjacent severity bands or absorbed residual covariance rather than adding a substantively new profile shape. The retained profiles were descriptively compared regarding total score with one-way analyses of variance. These comparisons were treated as descriptive summaries of expected alignment with the operational total score rather than as independent external validation.

To answer the second research question on how evenly the 1540 cutoff captured the retained profiles, the cutoff was cross-tabulated with latent-profile membership to describe the cutoff capture rates across profile types. To answer the third research question on whether profile distributions varied across background variables, associations between profile membership and selected background variables were examined with chi-square tests and Cramer’s *V*. Because these background analyses were based on modal class assignments, they were treated as descriptive rather than as error-corrected three-step estimates. Finally, robustness checks re-estimated the retained numbers of profiles with tied, spherical, and full covariance structures to determine whether the substantive conclusions depended on the diagonal local-independence specification.

### 3.6. Ethical Considerations

Written parental or guardian consent was originally obtained as part of the standard MMCAT registration and nomination process at the time of testing. For the present study, the analyses used de-identified archival assessment records extracted for secondary research use. After direct identifiers were removed, the analytic file posed minimal privacy risk. Access to the data was authorized by the Education Administration in Al-Ahsa, and the secondary use of these records for this study was separately approved by the Research Ethics Committee at King Faisal University (ref. no KFU-2023-NOV-ETHICS1381).

## 4. Results

### 4.1. Preliminary Score Structure

Table 1 summarizes the score structure within each assessment level. Across levels, domain intercorrelations ranged from 0.40 to 0.66, whereas correlations between the domains and the total score ranged from 0.66 to 0.88. These values indicate that the total score tracked the domain scores closely, especially at Level 3. In practical terms, students who performed well in one domain usually also performed well in the other domains. This pattern is consistent with a strong common performance dimension, and the progression from Levels 1 to 3 suggests that cross-domain homogeneity appeared to be stronger at the higher assessment levels. Against that more homogeneous background, the mathematical–spatial elevation in the Level 3 very high profile is notable because it appears as a local differentiation within an otherwise tightly coupled score structure.

### 4.2. Research Question 1: Do the Data Show Hidden Cognitive Heterogeneity?

Table 2 presents the fit indices for the latent profile models estimated within each level. The classification quality for the retained solutions was acceptable, with APPA values ranging from 0.84 to 0.87. The retained solutions were selected by considering the statistical fit, classification quality, class size, interpretability, and parsimony together rather than relying on any single criterion. For Level 1, the three-profile solution had the lowest BIC (72,561.76). Although AIC decreased slightly with four profiles, the classification accuracy declined (APPA = 0.800 vs. 0.859), the minimum class size decreased from 309 to 178, and the additional profile mainly divided an existing profile into two adjacent severity levels rather than identifying a substantively new pattern. For Level 2, the BIC favored the four-profile solution by 14.77 points (88,227.21 vs. 88,241.98), which represents very strong evidence in favor of four profiles according to conventional guidelines (Kass & Raftery, 1995). However, the four-profile solution had clearly lower classification accuracy (APPA = 0.793 vs. 0.867) and a much smaller minimum class size (*n* = 154 vs. 457), and the additional profile did not identify a substantively new pattern. The three-profile solution was therefore retained because it provided better classification quality, a larger minimum class, and a more parsimonious representation of the data. For Level 3, the four-profile solution had the lowest BIC (80,648.06). Adding a fifth profile reduced the classification accuracy (APPA = 0.780 vs. 0.843) and the minimum class size from 216 to 171. In the scikit-learn solution, the additional class subdivided an existing high-performing profile rather than identifying a distinct profile shape.

**Figure 1:**
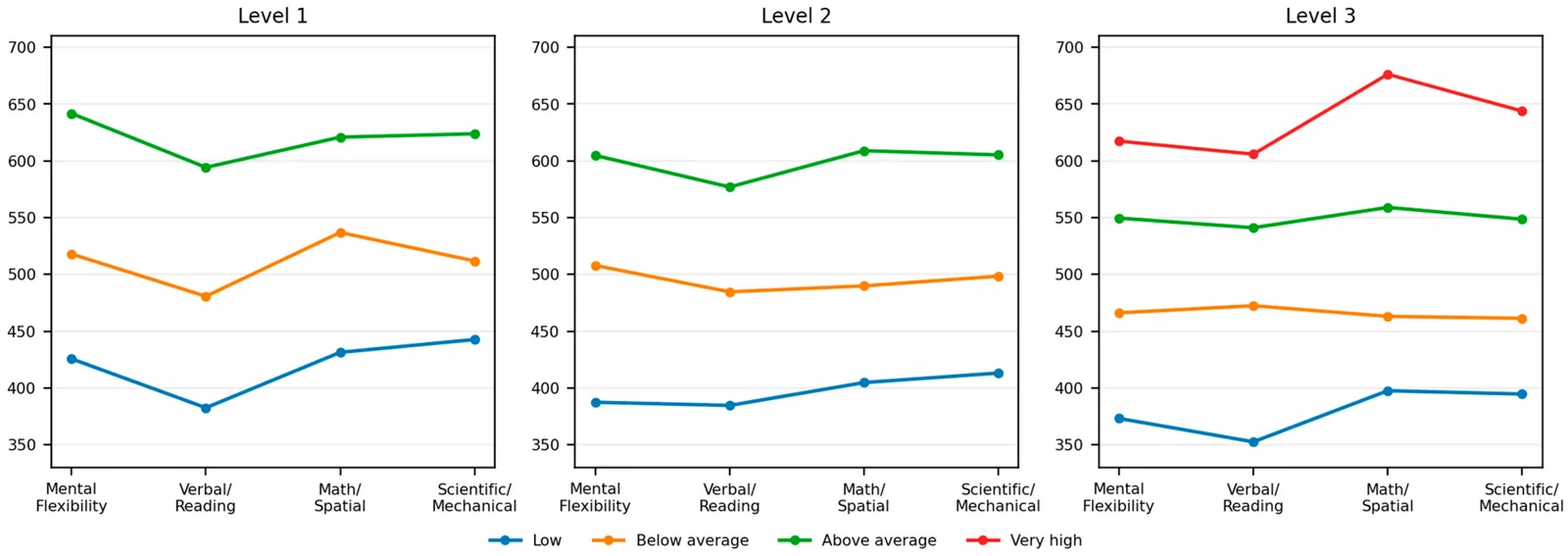
Latent profile plots by assessment level. Note: The largely parallel lines at Levels 1 and 2 indicate that the retained profiles differ mainly in the overall level of performance. Level 3 shows the clearest departure from strict parallelism, with a stronger mathematical–spatial peak in the very high profile.

Figure 1 provides the clearest answer to the first research question. At Levels 1 and 2, the profile lines were largely parallel. This means that the main difference between the groups was the overall level of performance rather than sharply different patterns of strengths and weaknesses. Level 3 showed the same general pattern, but with one notable deviation: the very high profile displayed a clearer peak in mathematical–spatial reasoning than in the other domains. Even so, the overall pattern remained much more ordinal than spiky.

Robustness checks supported the same main interpretation. Table 3 summarizes the retained numbers of profiles re-estimated under alternative covariance structures. Supplementary models with tied and spherical covariance structures reproduced the same ordered profile pattern at Levels 1 and 2 and preserved the stronger mathematical–spatial elevation in the highest Level 3 profile. More flexible full-covariance mixtures improved the information criteria, as expected, but they also introduced covariance-driven splits and reduced the clarity of the ordered level structure, especially at Level 3. Because the study sought parsimonious local-independence profiles rather than unrestricted covariance mixtures, the diagonal covariance solutions were retained as the primary models.

Taken together, the results show that hidden heterogeneity was present, but it was mostly heterogeneity of level, not strongly differentiated cognitive shape. In other words, the retained profiles looked more like ordered performance strata than sharply distinct cognitive types.

### 4.3. Research Question 2: How Evenly Does the 1540 Cutoff Capture the Retained Profiles?

The next question was whether the 1540 cutoff score captured all retained profiles equally well. The answer was no. As expected, profile membership was strongly related to the operational total score within each level: Level 1, *F*(2, 1536) = 1673.19, *p* < .001, and *η*^2^ = 0.69; Level 2, *F*(2, 1872) = 2983.15, *p* < .001, and *η*^2^ = 0.76; and Level 3, *F*(3, 1728) = 5149.12, *p* < .001, and *η*^2^ = 0.90.

These extremely large *η*^2^ values are partly expected because the total score is derived from the same domain scores used to estimate the profiles. This creates some circularity in comparison. The results are therefore reported descriptively and should not be interpreted as independent evidence of external validity.

At Level 1, the cutoff captured 80.58% of the high profile but only 15.38% of the average profile (Table 4). Thus, most students in the average profile remained below the administrative threshold despite sharing the same broad profile pattern.

Level 2 showed the same basic pattern (Table 5). The cutoff captured 91.25% of the high profile but only 15.48% of the average profile. Again, the rule captured globally high performers much more often and was much more selective around the middle range.

Level 3 also showed strong differentiation in cutoff capture (Table 6). None of the low or below-average profiles met the cutoff, 79.28% of the above-average profiles met it, and all students in the very high profile met it. This level also showed the clearest sign of profile-shape differentiation because the very high profile displayed a stronger mathematical-spatial peak than the other profiles.

These results show that the 1540 cutoff captured globally high profiles much more often than adjacent middle-range profiles. The most important point is that students with the same broad cognitive pattern could receive different decisions if they fell near the cutoff. This was most visible at Levels 1 and 2, where the average profile was split into a smaller above-cutoff subgroup and a much larger below-cutoff subgroup. The current rule therefore appears to privilege broad high-level performance and to be less inclusive of adjacent middle-range profiles.

The same tables also show that students who met the cutoff were not drawn from only one latent profile. At Level 1, identified students came from the average profile (*n* = 112) and high profile (*n* = 249). At Level 2, they came from the average profile (*n* = 135) and high profile (*n* = 417). At Level 3, they came from the above-average profile (*n* = 394) and very high profile (*n* = 216). Thus, even after a single cutoff was applied, the identified group was not fully homogeneous.

### 4.4. Research Question 3: Do Profile Distributions Vary Across Background Variables?

Background-variable influences were mostly small in magnitude, especially at Levels 1 and 2. Table 7 summarizes Cramer’s *V* for gender, school type, test language, and grade within each level. The clearest demographic pattern was gender at Level 3 (*χ*^2^(3, *N* = 1732) = 54.13, *p* < .001, *V* = 0.18). School type at Level 3 also showed a nontrivial association (*V* = 0.15), whereas language effects were small at all levels. Grade effects were expected at Levels 1 and 2 because developmental progression is partly built into the level structure, and thus, they are reported descriptively rather than interpreted as independent explanatory effects.

Table 8 shows the Level 3 gender pattern in more concrete terms. Male students made up 28.0% of the low profile, 28.1% of the below-average profile, 35.6% of the above-average profile, and 53.7% of the very high profile. This was the clearest background pattern in the dataset. It does not, by itself, explain why the Level 3 profiles differ, but it shows that the highest profile at the upper level was more male-heavy than the lower profiles.

In general, the revised analyses support three main conclusions. First, the retained profile solutions were mainly ordinal rather than sharply shape-based. Second, the 1540 cutoff was not neutral across profiles: it captured globally high performers much more often and was much more selective around the middle range. Third, the clearest background difference appeared at Level 3, especially for gender. Together, these findings suggest that the present identification rule captures broad high-level performance efficiently, but it does not represent all adjacent profile types equally.

## 5. Discussion

The present study examined whether the use of a single total-score cutoff in the Mawhiba system may hide meaningful differences in students’ cognitive profiles. The findings suggest one central point: a single cutoff may be efficient for administrative decisions, but it can also compress meaningful variation between students who are close to the decision boundary. In this study, the retained profile solutions were meaningful, but they were mainly ordinal rather than sharply different shapes. At the same time, the 1540 cutoff did not capture all profile types equally well. It captured students with broadly high performance much more often, but it was much more selective for students in the middle range. This means that the main issue is not only who is identified as gifted but also what kind of cognitive pattern is being recognized and what kind of educational response may be needed next. In this sense, the present findings have implications not only for identification policy but also for programming decisions after identification (Peters et al., 2023; Subotnik et al., 2011).

The first important implication is that performance on the MMCAT appears to be strongly organized by a common performance dimension. This interpretation is supported by the high correlations between the four domains and the total score, and by the largely parallel profile lines at Levels 1 and 2. In simple terms, students who performed well in one domain usually also performed well in the others. This means that the assessment does not show a highly fragmented structure of separate abilities. Instead, it seems to capture a broad dimension of strong cognitive performance, with only limited profile shape differences. This interpretation fits well with talent development views that distinguish between broad early potential and later domain-specific pathways of excellence (Subotnik et al., 2011).

At the same time, the present findings do not support a strong version of Spearman’s law of diminishing returns. That line of work suggests that cognitive abilities may become less strongly related at higher levels of ability (Blum & Holling, 2017). In the present cross-sectional, level-stratified data, however, the pattern ran in the opposite direction: domain correlations were somewhat higher at Level 3 than at Level 1. Because the design is not longitudinal, this should be interpreted as a difference across assessment levels in this screened sample rather than as direct evidence of developmental change. This does not mean that specialization is absent; rather, it suggests that specialization may begin to appear within a still strongly connected cognitive structure. This helps explain why the very high profile at Level 3 remained elevated across all domains while also showing a clearer mathematical–spatial peak.

Taken together, the high domain–total correlations, the somewhat stronger cross-domain homogeneity observed at the higher assessment levels, and the largely parallel latent profiles suggest that differentiation in the present sample was local rather than global. In other words, the mathematical–spatial elevation in the very high Level 3 profile appeared within an otherwise strongly shared performance structure, not as evidence of a broad separation between abilities. This pattern is more consistent with a moderate reading of differentiation theory than with a strong version of Spearman’s law of diminishing returns (Blum & Holling, 2017). At the same time, it also fits talent development views suggesting that broad early ability may remain highly integrated, even when more specific domain strengths begin to emerge (Subotnik et al., 2011).

The choice of a person-centered method is also important for interpreting these findings. Latent profile analysis was selected because it allows researchers to identify hidden heterogeneity in multidimensional data and to develop models iteratively by combining statistical and substantive criteria (Berlin et al., 2014). This is especially relevant in gifted education, where researchers have argued that gifted students should not be treated as one uniform group. Work using latent profile analysis in gifted education has shown that person-centered methods can provide a more nuanced understanding of variability between advanced learners than variable-centered approaches alone (Mammadov et al., 2016). The present study supports this point, but with an important qualification: the retained mixture solutions summarized meaningful heterogeneity, yet that heterogeneity was mostly a matter of level rather than a sharply different profile shape.

The largely ordinal profiles do not weaken the rationale for using LPA. Whether students differed mainly in overall performance level or in the shape of their cognitive profiles was an empirical question in this study. Variable-centered analyses can describe associations between cognitive domains, but they do not directly test whether subgroups of students show different configurations of strengths and weaknesses. LPA allowed us to examine this possibility without assuming in advance that the profiles would differ in level, shape, or both. At Levels 1 and 2, the retained profiles differed mainly in overall performance, with little evidence of distinct profile shapes. This finding suggests that heterogeneity at these levels was better represented by broad performance levels than by different configurations across cognitive domains. At Level 3, the highest-performing profile showed some differentiation in mathematical–spatial reasoning. This result should be interpreted cautiously because it may partly reflect differences in item design at this assessment level rather than developmental differences alone (see Section 6). LPA therefore helped determine how heterogeneity appeared in the data rather than assuming its form in advance. The cutoff analysis also showed the value of examining latent profiles. At Levels 1 and 2, only a minority of students in the average profile met the cutoff of 1540, while most remained below it. Students within the same broad profile could therefore fall on different sides of the cutoff. This finding shows that the cutoff divided students who had similar patterns of cognitive performance, which would not be apparent from the total-score classification alone.

The Level 3 pattern is especially important because it points to the educational value of spatial reasoning at more advanced levels. Long-term research by Lubinski, Benbow, and colleagues has shown that spatial ability is not a minor side skill. It is an important predictor of later success in STEM fields and one that has often been underused in talent identification systems (Lubinski, 2010; Wai et al., 2009). In this context, the mathematical-spatial elevation seen in the highest Level 3 profile may be more than a statistical detail. It may indicate a more specialized pattern that becomes clearer in adolescence, even while general performance remains high across domains. This distinct profile shape at Level 3 may reflect genuine cognitive differentiation during development, or it may be partly due to differences associated with the level-specific item forms. Different MMCAT forms are developed for each grade band and subsequently linked to place their items on a common scale. As the grade level and test form vary together by design, the present data cannot distinguish between these two explanations. Although later outcomes cannot be tested directly in this study, the observed pattern is consistent with the broader literature on the importance of spatial ability in advanced academic development (Lubinski, 2010; Wai et al., 2009).

A second major implication concerns the meaning of the 1540 cutoff. The present results suggest that a fixed cutoff should be understood as an administrative estimate rather than as an absolute truth. Standard-setting research has long shown that cut scores are policy decisions built on psychometric evidence and professional judgment, not natural boundaries that separate clearly different kinds of students (Cizek & Bunch, 2007). In addition, the broader testing standards literature makes clear that classification decisions near a cut score should be interpreted carefully, because every observed score contains some degree of measurement error and classification consistency is never perfect (AERA et al., 2014).

This concern is clearly relevant to the present results. At Levels 1 and 2, many students in the average profile shared the same broad cognitive pattern, yet only a minority crossed the 1540 threshold. This suggests that within the profiles identified in this study, small score differences near the cutoff may not always reflect meaningful differences in underlying cognitive performance. In practical terms, a student who scores just below the cutoff may not be cognitively different in an important way from a student who scores just above it if both belong to the same broad latent profile.

However, these findings should be interpreted at different levels. Psychometrically, the latent profiles describe patterns of cognitive performance and show that the operational cutoff does not capture all profiles at the same rate. Administratively, this does not mean that students below the cutoff should necessarily have been identified as gifted, because identification decisions depend on the criteria and purposes of the identification system. Educationally, the profiles may provide additional information about patterns of strengths and weaknesses that could be considered when planning educational support for gifted students. From a policy perspective, these findings suggest the potential value of considering score bands, borderline review procedures, or other decision rules alongside a single cutoff, particularly when score precision near the boundary is limited. The broader issue is not whether cutoffs are useful, but whether they are treated too rigidly when interpreting scores near the decision boundary (AERA et al., 2014; Cizek & Bunch, 2007; Peters et al., 2023).

The cutoff captured the high profile very well at Levels 1 and 2 and the very high profile completely at Level 3, but it was much more selective for the adjacent middle-range profiles. This pattern should not be interpreted primarily as evidence that the 1540 cutoff is poorly set or technically flawed; rather, uneven capture across profiles is expected to some extent when a single total-score threshold is used, because students with higher overall performance are more likely to meet the cutoff. At the same time, this pattern shows why profile-based information may be useful alongside the total score. A single summary score does not show whether differences between students reflect the overall performance level, profile shape, or both. The profiles provide additional information about these differences within the screened sample. Therefore, the total-score cutoff should be interpreted cautiously and should not be treated as the only source of information about students’ cognitive performance. In this sense, profile-based interpretation may supplement the current identification system rather than replace the operational cutoff. This interpretation fits with recent work arguing that identification systems should be judged not only by simplicity but also by their sensitivity and alignment with their intended purposes (Peters et al., 2023).

A related point is that the present study speaks more strongly to classification after screening than to access to screening itself. These data came from students who had already moved through a school-based nomination pathway before taking the MMCAT. Therefore, the study is strongest in showing how multidimensional performance is treated once students reach the testing stage. This distinction is important internationally. Card and Giuliano (2016) showed that universal screening can substantially increase the representation of low-income and minority students in gifted education, even when the eligibility standard itself does not change. Taken together, the literature and the present findings suggest that equitable identification has at least two stages: fair access to assessment and fair interpretation of multidimensional performance once assessment occurs (Card & Giuliano, 2016; Peters et al., 2023). This nomination pathway also has an important consequence for the present analysis. Because the sample is restricted toward the upper end of the ability range, the retained profiles may understate the degree of shape-based heterogeneity that could be observed in a broader, non-nominated sample.

The present findings are also meaningful in the Saudi and Arab contexts. Earlier work by Aljughaiman and Ayoub (2017) showed that nomination and identification practices in Arabic environments may favor some forms of giftedness more than others and may under-recognize less visible forms of giftedness. The current findings extend that concern. They show that even after students enter the formal testing stage, a single composite cutoff may still capture some nearby profile types less often than others. This gives the study local and international relevance: the problem is not only who becomes nominated but also how multidimensional performance is reduced to one final decision.

The finding that students above the cutoff came from more than one retained profile also has practical meaning. Even after the 1540 rule was applied, the identified group was not fully homogeneous. This means that gifted students are not a monolith. Some identified students seem to represent broad, globally high performance, whereas others come from profiles that are closer to the middle range but still pass the threshold. This finding leads to what may be called the identification–programming gap. A single cutoff may be administratively efficient, but it compresses multidimensional performance into one decision point. As a result, students from profiles that differ mainly in their overall performance level may be treated as a single group, while differences in how the operational cutoff captures these profiles may be overlooked. From a talent development perspective, identification should not only answer who qualifies for services, but should also help answer what kind of programming each student is likely to need (Subotnik et al., 2011).

This point has direct implications for practice. A student with broad high performance may be well served by a general enrichment model that offers advanced challenge across several domains. By contrast, a profile with clearer mathematical–spatial elevation, such as the very high profile at Level 3, may benefit more from specialized opportunities in engineering, technical design, robotics, advanced mathematics, or other spatially demanding learning experiences. This is where the present study moves beyond the narrow question of “Who is gifted?” toward a more educationally useful question: What kind of gifted learner is this, and what kind of opportunity does this learner need next? This shift is also consistent with earlier Saudi research showing that carefully designed enrichment can strengthen advanced abilities among gifted students (Aljughaiman & Ayoub, 2012).

The gender pattern at Level 3 also deserves careful attention. In this study, the clearest background difference appeared at the highest level, where males were more strongly represented in the very high profile than in the lower profiles. This same profile also showed the clearest mathematical–spatial elevation. In the Saudi context, this pattern may reflect differences in educational exposure, interests, extracurricular opportunities, or pathways into mathematically and technically oriented activities during adolescence. At the same time, the pattern is broadly consistent with prior research showing that gender differences in spatial reasoning tend to become clearer with age and may remain visible, even in highly selected samples (Lauer et al., 2019; Tsigeman et al., 2023). These findings should be considered alongside—rather than given precedence over—educational, cultural, and exposure-related explanations. The present data cannot test these explanations directly. Therefore, the safest conclusion is that the pattern should be reported clearly and studied further but not treated as explanatory or causal.

Because the clearest background difference appeared at Level 3, especially for gender, future research should also examine measurement invariance across male and female students before stronger conclusions are drawn about substantive gender differences in profile structure or representation. As Vandenberg and Lance (2000) argued, group comparisons are more defensible when there is evidence that the same construct is being measured in the same way across groups. The present study could not test invariance directly because item-level responses were not available, but this should be a priority for future work. More broadly, with the available information, we could not confirm whether the domain scores were directly comparable across school type or test language. Therefore, the demographic differences observed in this study should be interpreted cautiously rather than as confirmed group differences.

## 6. Limitations

This study has several limitations. First, the analyses were based on archival administrative data rather than item-level responses. Because of this, the study could not re-estimate reliability, examine item functioning, test measurement invariance directly, or fit item-level measurement models. In addition, because the technical manual and item-level data were not available, the study could not examine how the standardized domain scores were constructed or whether they were directly comparable across assessment levels, gender, school type, or test language. Conducting the profile analyses separately within each assessment level reduced concerns about comparisons across levels. However, comparability across gender, school type, and test language should be examined in future research.

Second, the mixture models were estimated using scikit-learn for consistency with the Python-based analytic pipeline. However, scikit-learn does not provide the LMR or BLRT commonly used to compare adjacent profile solutions. As an additional check, we re-estimated the retained solutions and the adjacent more complex solutions using StepMix, which implements the BLRT. The results were mixed. The BLRT supported three profiles at Level 1 but favored an additional profile at Levels 2 and 3. At Level 2, the additional profile mainly subdivided an existing performance pattern and was accompanied by a lower APPA and a much smaller minimum class. The Level 3 result was less clear. The five-profile solution had a lower APPA but also a lower BIC and higher entropy, and its additional class (*n* = 66) showed a potentially distinct profile shape. Because this class was small, its stability and generalizability remain uncertain. We retained the four-profile solution for the primary analyses but acknowledge that this decision is not conclusive and should be examined in larger or independent samples. The retained solutions were not additionally cross-validated using an R-based implementation such as mclust or tidyLPA.

Third, because item-level responses were not available, measurement invariance across genders could not be tested directly, which limits the interpretability of the gender comparisons reported in Table 8.

Fourth, the data came from a screened administrative sample rather than a random draw from the full student population in Al-Ahsa. Because students entered through school nomination, the sample is truncated toward the upper end of the ability range. This restriction may compress shape-based variation and make level-based differences more prominent. It may also reduce the number of latent profiles that can be recovered because students at the extremes of the broader ability distribution, where distinct profiles may be more likely to emerge, are largely absent from the data. Level 3 still showed a distinct profile shape despite this restriction, suggesting that the LPA could detect shape heterogeneity when it was sufficiently pronounced. However, additional latent profiles or more pronounced shape differences may have remained undetected at Levels 1 and 2 because of the restricted ability range. Testing this possibility would require a broader, non-nominated sample spanning the full ability range.

Fifth, the available data were limited to demographic variables and assessment scores. They did not include other important information such as teacher ratings, family background, later academic achievement, participation in enrichment programs, or longitudinal follow-up data. For this reason, this study could not test whether the retained profiles differed in later outcomes.

Sixth, the background-variable analyses were descriptive because they were based on modal class assignment rather than error-corrected three-step methods.

Finally, this study used cross-sectional data from one screening cycle only. Therefore, the findings should be interpreted as describing patterns within this assessment context rather than as direct evidence of long-term developmental pathways.

## 7. Conclusions

In conclusion, the present study has value beyond the Saudi context. Many countries use staged systems in which students first enter a nomination or referral pathway and are then classified using formal measures. The present findings show that even when a multidimensional assessment is used, a single total-score cutoff may not fully reflect the variation in students’ cognitive performance profiles. In this study, the cutoff worked efficiently for identifying broadly high performers, but it did not capture all retained profiles at the same rate. The broader lesson is not that identification systems should abandon summary scores; rather, identification systems should balance efficiency with careful interpretation of scores near the cutoff. In the present study, profile-level interpretation provided complementary information about variation in cognitive performance that was not reflected in the total-score classification alone. Summary scores are useful, but they should not be the only lens through which multidimensional talent is interpreted (AERA et al., 2014; Peters et al., 2023; Subotnik et al., 2011).

## Figures and Tables

**Table 1:** Descriptive statistics and correlation ranges by assessment level.

Level	*n*	Mental Flexibility M (*SD*)	Verbal/Reading M (*SD*)	Math/Spatial M (*SD*)	Scientific/Mechanical M (*SD*)	Total Score M (*SD*)	Domain Intercorr. Range	Domain–Total Corr. Range
Level 1	1539	512.63 (105.57)	471.47 (100.36)	519.31 (92.86)	511.72 (100.44)	1473.04 (91.50)	0.40–0.53	0.66–0.73
Level 2	1875	496.37 (105.74)	478.00 (96.63)	494.07 (98.38)	499.58 (103.12)	1482.51 (99.65)	0.49–0.55	0.74–0.79
Level 3	1732	487.59 (93.64)	481.26 (108.13)	502.12 (109.81)	493.80 (98.51)	1498.66 (102.99)	0.54–0.66	0.82–0.88

Note: The domain intercorrelation range reports the smallest and largest correlations between the four domain scores within each level. The domain-total correlation range reports the smallest and largest correlations between the domain scores and the operational total score.

**Table 2:** Model fit indices for level-stratified latent profile solutions.

Level	Profiles	AIC	BIC	Entropy	APPA	Smallest Class *n*	Retained
Level 1	1	74,144.27	74,186.98	—	—	1539	
Level 1	2	72,690.14	72,780.90	0.740	0.922	636	
Level 1	3	72,422.95	72,561.76	0.697	0.859	309	Yes
Level 1	4	72,420.70	72,607.56	0.657	0.800	178	
Level 1	5	72,416.26	72,651.18	0.584	0.725	207	
Level 1	6	72,416.68	72,699.64	0.598	0.716	174	
Level 2	1	90,508.17	90,552.46	—	—	1875	
Level 2	2	88,504.17	88,598.29	0.757	0.927	904	
Level 2	3	88,098.04	88,241.98	0.714	0.867	457	Yes
Level 2	4	88,033.44	88,227.21	0.655	0.793	154	
Level 2	5	88,028.60	88,272.20	0.627	0.754	106	
Level 2	6	88,017.46	88,310.89	0.587	0.693	121	
Level 3	1	83,797.27	83,840.92	—	—	1732	
Level 3	2	81,255.05	81,347.82	0.814	0.944	800	
Level 3	3	80,605.41	80,747.29	0.787	0.902	446	
Level 3	4	80,457.07	80,648.06	0.734	0.843	216	Yes
Level 3	5	80,425.58	80,665.69	0.682	0.780	171	
Level 3	6	80,406.73	80,695.95	0.646	0.736	158	

Note: Models were estimated within each level using Gaussian finite-mixture models with diagonal covariance matrices. Retained solutions were chosen based on the AIC, BIC, entropy, APPA, smallest class size, substantive interpretability, and parsimony. All models were re-estimated from 100 k-means-based starting values, and the highest log-likelihood solution was retained.

**Table 3:** Robustness checks for retained profile numbers across alternative covariance structures.

Level	Diagonal BIC/APPA	Tied BIC/APPA	Spherical BIC/APPA	Full BIC/APPA	Conclusion
Level 1	72,561.76/0.859	71,798.27/0.842	72,552.33/0.859	71,882.33/0.838	Same ordered three-profile pattern. Full covariance improved the fit, but it mainly absorbed the covariance instead of adding a new shape.
Level 2	88,242.09/0.867	87,231.36/0.787	88,235.57/0.866	87,308.84/0.809	Same ordered three-profile pattern. More flexible structures lowered the classification sharpness without changing the main interpretation.
Level 3	80,648.06/0.843	79,704.70/0.799	80,718.02/0.839	79,855.51/0.796	The mathematical–spatial elevation in the highest profile remained. Full covariance added covariance-driven splits and weaker profile clarity.

Note: Entries report the BIC and APPA for the retained number of profiles in each level (3, 3, and 4). Across covariance structures, the main interpretation remained the same: Levels 1 and 2 showed ordered severity bands, and Level 3 retained the clearest mathematical–spatial elevation in the highest profile.

**Table 4:** Level 1: Latent profile characteristics and cutoff capture rates for the 1540 total score rule.

Profile	*n* (%)	Mental Flexibility	Verbal/ Reading	Math/ Spatial	Scientific/ Mechanical	Total Score	% ≥ 1540	% < 1540
Low	502 (32.6)	425.64	382.54	431.36	442.64	1381.65	0.00	100.00
Average	728 (47.3)	517.87	480.70	536.88	511.79	1484.64	15.38	84.62
High	309 (20.1)	641.64	594.19	620.80	623.79	1594.17	80.58	19.42

Note: Percentages in the final two columns indicate the proportions of students in each latent profile who met versus failed to meet the operational cutoff of 1540.

**Table 5:** Level 2: Latent profile characteristics and cutoff capture rates for the 1540 total score rule.

Profile	*n* (%)	Mental Flexibility	Verbal/ Reading	Math/ Spatial	Scientific/ Mechanical	Total Score	% ≥ 1540	% < 1540
Low	546 (29.1)	387.42	384.61	404.74	413.11	1370.33	0.00	100.00
Average	872 (46.5)	507.80	484.62	489.85	498.40	1486.61	15.48	84.52
High	457 (24.4)	604.72	576.92	608.86	605.16	1608.71	91.25	8.75

Note: Percentages in the final two columns indicate the proportions of students in each latent profile who met versus failed to meet the operational cutoff of 1540.

**Table 6:** Level 3: Latent profile characteristics and sensitivity of the 1540 total score cutoff.

Profile	*n* (%)	Mental Flexibility	Verbal/ Reading	Math/ Spatial	Scientific/ Mechanical	Total Score	% ≥ 1540	% < 1540
Low	397 (22.9)	373.15	352.55	397.55	394.64	1364.58	0.00	100.00
Below average	622 (35.9)	466.13	472.40	463.02	461.26	1468.61	0.00	100.00
Above average	497 (28.7)	549.50	541.04	558.94	548.55	1568.70	79.28	20.72
Very high	216 (12.5)	617.31	605.81	676.15	643.81	1670.48	100.00	0.00

Note: Percentages in the final two columns indicate the proportions of students in each latent profile who met versus failed to meet the operational cutoff of 1540.

**Table 7:** Association strengths between profile membership and selected background variables.

Level	Gender *V*	School Type *V*	Test Language *V*	Grade *V*
Level 1	0.053	0.081	0.047	0.286
Level 2	0.068	0.081	0.062	0.123
Level 3	0.177	0.148	0.064	0.036

Note: Entries are Cramer’s *V* values computed within level. Background-variable analyses were descriptive because they were based on modal class assignment. The clearest background effect was gender at Level 3 (*χ*^2^(3, *N* = 1732) = 54.13, *p* < .001, *V* = 0.18). Grade effects are reported descriptively because grade is partly embedded in the level structure.

**Table 8:** Level 3: Gender composition of the retained latent profiles.

Profile	Female, *n* (%)	Male, *n* (%)
Low	286 (72.0)	111 (28.0)
Below average	447 (71.9)	175 (28.1)
Above average	320 (64.4)	177 (35.6)
Very high	100 (46.3)	116 (53.7)

Note: Percentages are calculated within profile.

## Data Availability

The data underlying this study are available from the corresponding author upon request; however, they are not publicly shared due to confidentiality and privacy considerations.
